# Cell culture: complications due to mechanical release of ATP and activation of purinoceptors

**DOI:** 10.1007/s00441-017-2618-8

**Published:** 2017-04-22

**Authors:** Geoffrey Burnstock, Gillian E. Knight

**Affiliations:** 1 Autonomic Neuroscience Centre, Royal Free and University College Medical School, Rowland Hill Street, London, NW3 2PF UK; 2 Department of Pharmacology and Therapeutics, The University of Melbourne, Melbourne, Australia

**Keywords:** P1 receptors, P2 receptors, Purinoceptor, Shear stress, Ectonucleotidases

## Abstract

There is abundant evidence that ATP (adenosine 5′-triphosphate) is released from a variety of cultured cells in response to mechanical stimulation. The release mechanism involved appears to be a combination of vesicular exocytosis and connexin and pannexin hemichannels. Purinergic receptors on cultured cells mediate both short-term purinergic signalling of secretion and long-term (trophic) signalling such as proliferation, migration, differentiation and apoptosis. We aim in this review to bring to the attention of non-purinergic researchers using tissue culture that the release of ATP in response to mechanical stress evoked by the unavoidable movement of the cells acting on functional purinergic receptors on the culture cells is likely to complicate the interpretation of their data.

## Introduction

While it was recognised early that ATP (adenosine 5′-triphosphate) is released from damaged or dying cells, it was shown more recently that gentle mechanical perturbation, such as shear stress, membrane stretch and hypo-osmotic cell swelling, leads to release of ATP from most cell types (Bodin and Burnstock 2001; Bodin et al. 1991; Chaudry 1982; Dolovcak et al. 2011; Forrester 1972; Grygorczyk and Guyot 2001; Milner et al. 1990, 1992; Praetorius and Leipziger 2009, 2010; Sperlágh et al. 2007; Wang et al. 1996). In the outstanding review by Lazarowski et al. (2011), it was stated that “P2Y receptor expression-dependent formation of second messengers was noted in cultured cells subjected to mechanical stress, for example medium displacement or cell wash (Filtz et al. 1994; Lazarowski et al. 1995; Parr et al. 1994). A vast number of studies have followed, illustrating that nonlytic release of ATP occurred in practically every cell type subjected to physical stresses, such as flow resulting in shear stress, hydrostatic pressure, osmotic swelling or shrinking, compressive stress, mechanical loading, plasma membrane stretch, hypoxia and cell swelling” performed during routine experimental procedures, such as cell rinsing and medium changes. It is unlikely that ATP release caused by gentle mechanical stimulation arises from cell damage, for example mechanical stimulated ATP release occurs without associated membrane conductive changes (Hamill and Martinac 2001). Many novel assays (or sensors) have been developed to detect ATP release from cells, including luciferin–luciferase bioluminescence and atomic force microscopy (see Dale and Frenguelli 2012; Furuya et al. 2014; Khlyntseva et al. 2009; Praetorius and Leipziger 2009).

The mechanisms responsible for the transport of ATP from cells have been a matter of intense debate. For most cell types, it appears to be a combination of vesicular exocytosis and connexin or pannexin hemichannels (Dahl 2015; Dubyak 2007; Lazarowski et al. 2011; Li et al. 2011; Lohman and Isakson 2014; Novak 2003; Scemes et al. 2009; Spray et al. 2006), although for some cells ATP-binding cassette transporters or maxi ion channels have been claimed (Sabirov and Okada 2005). It has also been proposed that P2X7 receptors may mediate ATP release (Pellegatti et al. 2005; Suadicani et al. 2006). A vesicular nucleotide transporter has been identified (Sawada et al. 2008).

ATP released from cells is rapidly broken down by ectonucleotidases to adenosine (see Cardoso et al. 2015; Yegutkin 2008; Zimmermann 2006) but both ATP and adenosine will have functional effects on the cells via P1, P2X and P2Y receptors (see Corriden and Insel 2010).

Two purinoceptor families were recognised in 1978, namely P1 (adenosine) and P2 (nucleotide) receptors (Burnstock 1978). Purinoceptor subtypes were cloned and characterised in the early 1990s, consisting in 4 P1 G protein-coupled receptor subtypes, 7 P2X ion channel receptor subtypes and 8 P2Y G protein-coupled receptor subtypes (see Burnstock 2007; Ralevic and Burnstock 1998).

## Release of ATP from cultured cells in response to mechanical stimulation

A comprehensive summary is shown in Table 1.

**Table 1:** ATP release from cultured cells in response to mechanical stimulation

Cell type	Stimulus	References
Vascular endothelial cells	Shear stress	Bodin et al. 1991
		Li et al. 2015
		Milner et al. 1990, 1992
		Xiang et al. 2007
		Yamamoto et al. 2011
	Hypotonic stress	Hisadome et al. 2002
		Oike et al. 2000
		Shinozuka et al. 2001
	Mechanical stretch	Hamada et al. 1998
Airways		
Lung epithelial cells	Stretch	Ramsingh et al. 2011
		Zhang et al. 2014
	Mechanical stress	Guyot and Hanrahan 2002
		Homolya et al. 2000
	Hypotonic stress	Okada et al. 2006
		Ransford et al. 2009
		Seminario-Vidal et al. 2011
Nasal epithelial cells	Mechanical stimulation	Watt et al. 1998
Tracheal epithelial cells	Hypotonic stress	Kawakami et al. 2004
Eye		
Retinal ganglion cells	Swelling	Xia et al. 2012
	Mechanical stretch	Xia et al. 2012
Retinal pigment cells	Hypertonic stress	Eldred et al. 2003
	Hypotonic stress	Mitchell 2001
		Reigada and Mitchell 2005
Retinal glial (Müller) cells	Hypo-osmotic swelling	Brückner et al. 2012
		Voigt et al. 2015
Lens	Hypertonic stress	Eldred et al. 2003
Ciliary epithelial cells	Hypotonic stress	Li et al. 2010
		Mitchell et al. 1998
Trabecular meshwork cells	Mechanical stress	Luna et al. 2009
	Swelling	Li et al. 2011, 2012
Corneal endothelial cells	Mechanical stimulation	Gomes et al. 2005
Liver		
Hepatocytes	Hypotonic cell swelling	Pafundo et al. 2008
Biliary epithelium (cholangiocytes)	Hypotonic cell swelling	Roman et al. 1999
		Sathe et al. 2011
	Shear stress	Woo et al. 2008, 2010
Glial cells		
Astrocytes	Hypotonic cell swelling	Beckel et al. 2014
		Darby et al. 2003
		Liu et al. 2008
	Mechanical stimulation	Beckel et al. 2014
		Lee et al. 2015
		Stout et al. 2002
		Zhang et al. 2008
Astrocytoma cells	Hypotonic stress	Blum et al. 2010
		Joseph et al. 2003
Microglia	Mechanical stimulation	Bennett et al. 2008
Bladder urothelial cells	Stretch	Mansfield and Hughes 2014
		Sun and Chai 2002
		Sun et al. 2001
	Mechanical stress	McLatchie and Fry 2015
	Hypotonic stimulation	Birder et al. 2003
Muscle		
Vascular smooth muscle	Mechanical stretch	Hamada et al. 1998
Bronchial smooth muscle	Mechanical stretch	Takahara et al. 2014
Cardiomyoctes	Mechanical stretch	Kim and Woo 2015
		Oishi et al. 2012
	Swelling	Dutta et al. 2004, 2008
Fibroblasts		
L929 fibroblasts	Shear stress	Grierson and Meldolesi 1995
Subepithelial fibroblasts	Mechanical stimulation	Furuya et al. 2005, 2014
		Murata et al. 2014
NIH/3T3 fibroblasts	Hypotonic shock	Boudreault and Grygorczyk 2002, 2004
Cardiac fibroblasts	Hypotonic stimulation	Lu et al. 2012
Bone		
Bone marrow stromal cells	Fluid flow (shear stress)	Riddle et al. 2007
Periodontal ligament	Mechanical stress	Ito et al. 2014
		Luckprom et al. 2010, 2011
		Wongkhantee et al. 2008
Osteoblastic cells	Mechanical stress	Hecht et al. 2013
		Romanello et al. 2001, 2005
	Shear stress/fluid flow	Gardinier et al. 2014
		Genetos et al. 2005
		Rumney et al. 2012
		Xing et al. 2014
Intervertebral disc annulus cells	Vibratory stimulation	Yamazaki et al. 2003
Chondrocytes	Hypotonic challenge	Rosenthal et al. 2013
	Mechanical stress	Graff et al. 2000
		Kono et al. 2006
		Millward-Sadler et al. 2004
MLO-Y4 osteocytes	Mechanical loading by fluid flow	Genetos et al. 2007
	Focal-force stimulation	Wu et al. 2013
	Mechanical stimulation	Kringelbach et al. 2015
	Membrane stretch	Thompson et al. 2011
Immune cells		
Jurkat T lymphocytes	Hypertonic stress	Loomis et al. 2003
		Woehrle et al. 2010
		Yip et al. 2007
	Mechanical stress	Loomis et al. 2003
	Shockwaves	Weihs et al. 2014
		Yu et al. 2010
	Osmotic stress	Corriden et al. 2007
B lymphoblasts	Slow motion	Sakowicz-Burkiewicz et al. 2010
Neutrophils	Hypertonic stress	Chen et al. 2004, 2015
Mast cells	Hypo-osmotic stress	Wang et al. 2013
Macrophages	Hypotonic stress	Burow et al. 2015
Tumour cells		
Prostate cancer cells	Hypotonic stress	Nandigama et al. 2006
	Mechanical stress	Sauer et al. 2000
Hepatoma cells	Hypotonic stress	Dolovcak et al. 2011
		Espelt et al. 2013
		Feranchak et al. 2010
		Wang et al. 1996
Cholangiocarcinoma	Hypotonic cell swelling	Gatof et al. 2004
		Roman et al. 1999
Lung epithelial carcinoma (A549) cells	Hypotonic shock	Seminario-Vidal et al. 2011 Tatur et al. 2008
	Shear stress	Ramsingh et al. 2011
	Stretch	Grygorczyk et al. 2013
Mammary carcinoma (C127) cells	Hypotonic challenge	Hazama et al. 2000 Sabirov et al. 2001
Ehrlich ascites tumour cells	Mechanical stress	Pedersen et al. 1999
Ovarian carcinoma (SKOV-3) cells	Mechanical stimulation	Vázquez-Cuevas et al. 2014
L929 fibrosarcoma cells	Hypotonic challenge	Islam et al. 2012
Skin		
Adipose tissue-derived stem cells	Shock wave treatment	Weihs et al. 2014
Keratinocyte cell lines	Air stimulated	Denda and Denda 2007 Barr et al. 2013
	Mechanical stimulation	Burrell et al. 2005
		Koizumi et al. 2004
Pancreas		
Acinar cells	Mechanical stimulation	Haanes et al. 2014
Duct cells	Mechanical & hypotonic stress	Kowal et al. 2015
*Xenopus* oocytes	Hypertonic stress	Aleu et al. 2003
Stem cells		
Mesenchymal stem cells	Shock waves	Sun et al. 2013
		Weihs et al. 2014
Gut		
Epithelial cell lines	Hypotonic challenge	Dezaki et al. 2000
		van der Wijk et al. 2003
	Osmotic cell swelling	Tomassen et al. 2004
Salivary glands		
Submandibular gland	Mechanical stimulation	Ryu et al. 2010
Kidney		
Collecting duct epithelial cells	Mechanical stimulation	Hovater et al. 2008
A6 distal nephron epithelial cells	Mechanical stretch	Ma et al. 2002
	Hypotonic treatment	Gheorghiu and Van Driessche 2004
		Jans et al. 2002
		Silva and Garvin 2008
MDCK cells	Pressure pulses	Praetorius et al. 2005
	Shear stress	Rodat-Despoix et al. 2013
Epithelia from cysts of polycystic kidneys	Hypotonic challenge	Wilson et al. 1999
Blood cells		
Erythrocytes	Hypotonic stretch	Locovei et al. 2006
Platelets	Shear stress	Mills et al. 1968
Leukocytes	Osmotic stress	Corriden et al. 2007

## Purinergic receptor expression in cultured cells

A comprehensive summary is shown in Table 2.

**Table 2:** Purinergic receptor expression in cultured cells (references in Table 1)

Cell type	Receptors expressed		
	P2X	P2Y	P1
Vascular endothelial cells	P2X4, P2X5, P2X7	P2Y_1,2 and 12_	A_1_
Airways			
Lung epithelial cells	P2X4, P2X5	P2Y_1,2,4,6 and 11_	A_1_, A_2A_, A_2B_
Nasal epithelial cells		P2Y_2_, P2Y_6_, P2Y_11_	A_2B_
Tracheal epithelial cells	P2X4, P2X7	P2Y_1_, P2Y_2_	A_2B_
Eye			
Retinal ganglion cells	P2X2-7		A_1_, A_2A_, A_3_
Retinal pigment cells	P2X2, P2X3, P2X7	P2Y_2_	A_1_, A_2A_, A_2B_, A_3_
Retinal glial (Müller) cells	P2X7	P2Y_1_	A_1_
Lens	P2X1, P2X4		A_1_
Ciliary epithelial cells	P2X2, P2X3, P2X7	P2Y_2_	A_1_, A_2A_, A_2B_, A_3_
Trabecular meshwork cells	P2X1, P2X7		A_1_
Corneal endothelial cells	P2X4-7	P2Y_1,2,4 and 6_	
Liver			
Hepatocytes	P2X4, P2X7	P2Y_1,2,4 and 6_	A_2A_, A_2B_, A_3_
Biliary epithelium (cholangiocytes)	P2X4	P2Y_1,2,4,6,11,12 and 13_	A_2A_
Glial cells			
Astrocytes	P2X4, P2X7	P2Y_1_, P2Y_2_	A_1_, A_2A_, A_3_
Astrocytoma cells	P2X7	P2Y_1_, P2Y_2_	A_2A_, A_2B_, A_3_
Microglia	P2X4, P2X7	P2Y_1_, P2Y_11_, P2Y_12_	A_1_, A_2A_, A_2B_
Bladder urothelial cells	P2X2, P2X3, P2X4	P2Y_1,2,4 and 6_	A_1_
Muscle			
Vascular smooth muscle	P2X1, P2X2, P2X4	P2Y_1,2,4 and 6_	A_2A_, A_2B_, A_3_
Bladder smooth muscle	P2X1, P2X2	P2Y_2_, P2Y_6_	A_1_, A_2A_, A_2B_
Cardiomyoctes	P2X1,3,4,5,6 and 7	P2Y_1_, P2Y_2_	A_1_, A_2A_, A_2B_
Fibroblasts			
Fibroblasts	P2X7	P2Y_2_	A_2A_, A_2B_
Cardiac fibroblasts	P2X4, P2X7	P2Y_2_	A_1_, A_2A_, A_2B_, A_3_
Bone			
Bone marrow stromal cells	P2X7	P2Y_1,2,6 and 11_	A_2B_
Periodontal ligament		P2Y_1,2,4 and 6_	A_2A_
Osteoblastic cells	P2X1-7	P2Y_1,2,4,6,12,13 and 14_	A_2A_, A_2B_
Intervertebral disc annulus cells	P2X4, P2X7		
Chondrocytes	P2X1,3,4,5 and 7	P2Y_2_	A_2A_, A_2B_
MLO-Y4 osteocytes	P2X1,2,3,4 and 7	P2Y_2,4,12 and 13_	
Immune cells			
Jurkat T lymphocytes	P2X1,4,5 and 7		A_1_, A_2A_, A_2B_, A_3_
B lymphoblasts			A_2A_
Neutrophils	P2X1, P2X4, P2X7	P2Y_2,4,6 and 11_	A_1_, A_2A_, A_2B_, A_3_
Mast cells	P2X7	P2Y_1_, P2Y_2_	A_2A_, A_2B_, A_3_
Macrophages	P2X7	P2Y_2_, P2Y_6_	A_2A_, A_2B_
Tumour cells			
Prostate cancer cells	P2X4-7	P2Y_1,2,6 and 11_	A_1_, A_2A_, A_2B_, A_3_
Hepatoma cells		P2Y_1,2,4,6 and 13_	A_2A_, A_2B_, A_3_
Cholangiocarcinoma		P2Y_2_	
Lung epithelial carcinoma (A549) cells	P2X4-7	P2Y_2_, P2Y_4_, P2Y_6_	A_2A_, A_2B_, A_3_
Mammary carcinoma cells	P2X7	P2Y_1_	A_1_, A_2A_, A_3_
Ehrlich ascites tumour cells		P2Y_1_, P2Y_2_	
Ovarian carcinoma (SKOV-3) cells	P2X7	P2Y_2_, P2Y_6_	
L929 fibrosarcoma cells	P2X7		
Skin			
Keratinocyte cell lines	P2X2,3,5 and 7	P2Y_1,2,4,6 and 11_	
Pancreas			
Acinar cells	P2X12,3,4,6 and 7	P2Y_1,2,4,11,12,13 and 14_	A_1_, A_2A_, A_2B_
Duct cells	P2X1,2,4,5,6 and 7	P2Y_1,2,4,6,11,12,13 and 14_	A_1_, A_2A_, A_2B_, A_3_
*Xenopus* oocytes	P2X4	P2Y_2_-like	Atypical A_1_
Stem cells			
Mesenchymal stem cells	P2X4,5,6 and 7	P2Y_1,2,4,11,13 and 14_	A_1_, A_2A_, A_2B_
Gut			
Epithelial cell lines	P2X7	P2Y_2_, P2Y_6_	A_2A_, A_2B_
Salivary glands			
Submandibular gland	P2X1-7	P2Y_1_, P2Y_2_	
Kidney			
Collecting duct epithelial cells	P2X4, P2X5, P2X6	P2Y_1,2,4 and 6_	A_1_, A_2A_, A_2B_, A_3_
A6 distal nephron epithelial cells	P2X4	P2Y_1,_ P2Y_2_	A_1_, A_2_
MDCK cells	P2X7	P2Y_1,2,6 and 11_	A_1_
Epithelia from cysts of polycystic kidneys	P2X4, P2X5	P2Y_1_, P2Y_2,_ P2Y_6_	
Blood cells			
Erythrocytes	P2X1, P2X4, P2X7	P2Y_1_, P2Y_2_	A_2B_
Platelets	P2X1	P2Y_1_, P2Y_12_, P2Y_14_	A_2A_, A_2B_
Leukocytes	P2X4, P2X7	P2Y_2_, P2Y_6_	A_1_, A_2A_, A_2B_, A_3_

When cells are cultured, they de-differentiate, which is associated with changes in receptor expression. If the cell density is high, the cells usually re-differentiate and this again is associated with changes in receptor expression (see, e.g., Chamley et al. 1974). Upregulation of P2Y_2_ receptors in rat salivary gland cells during short-term culture has also been reported (Turner et al. 1997).

## Function of purinergic receptors on cultured cells in response to released ATP

A comprehensive review of the functional expression of P2 receptors on a wide range of cell types is available (Burnstock and Knight 2004). Some examples follow. ATP released from retinal epithelial cells acts via P2 receptors to increase the rate of fluid transport or decrease phagocytosis (Mitchell 2001) and regulate neural retinal progenitor cell proliferation (Pearson et al. 2005). ATP released by osteoblasts inhibits bone mineralisation (Orriss et al. 2013). Stretch-released ATP from fibroblasts results in cell proliferation (Wang et al. 2005). ATP released from astrocytes mediates glial calcium waves (Guthrie et al. 1999). ATP released from endothelial cells by shear stress acts on endothelial P2 receptors to release nitric oxide resulting in vasodilatation (Burnstock and Ralevic 2014).

Mechanically-induced Ca^2+^ waves have been observed in a variety of cells, including chondrocytes (D’Andrea and Vittur 1996), airways epithelial cells (Boitano et al. 1994; Hansen et al. 1993; Sanderson et al. 1990), glial cells, including Müller cells (Charles et al. 1991, 1992, 1993; Newman 2001), keratinocytes (Koizumi et al. 2004), endothelial cells (Demer et al. 1993), T cells (Wang et al. 2014), mast cells (Osipchuk and Cahalan 1992) and others (see Leybaert and Sanderson 2012). It is likely that they are due to the activation of purinergic receptors by ATP released from the mechanically stimulated cells, mainly via P2Y_1_ and P2Y_4_ receptors (Frame and de Feijter 1997; Gallagher and Salter 2003; Stamatakis and Mantzaris 2006). Calcium waves are a dynamic intracellular signalling mechanism that allows spatiotemporal information to be rapidly propagated in tissues. ATP released at sites of cell stress signals danger to the immune system.

## Conclusion: need for re-interpretation of data derived from cell culture experiments

Release of ATP from cultured cells is unavoidable, due to gentle mechanical stimulation. The released ATP acts on purinoceptors expressed by these cells, which mediate both secretion and trophic events, such as cell proliferation, differentiation, death and migration. These events mean that interpreting results from experiments based on tissue culture need to take into account the effects of released ATP and its actions on purinoceptors.
